# Giant spontaneous exchange bias triggered by crossover of superspin glass in Sb-doped Ni_50_Mn_38_Ga_12_ Heusler alloys

**DOI:** 10.1038/srep30801

**Published:** 2016-08-01

**Authors:** Fanghua Tian, Kaiyan Cao, Yin Zhang, Yuyang Zeng, Rui Zhang, Tieyan Chang, Chao Zhou, Minwei Xu, Xiaoping Song, Sen Yang

**Affiliations:** 1 School of Science, MOE Key Laboratory for Nonequilibrium Synthesis and Modulation of Condensed Matter, Xi’an Jiaotong University, Xi’an 710049, China

## Abstract

A spontaneous exchange bias (SEB) discovered by Wang *et al*. [Phys. Rev. Lett. 106 (2011) 077203.] after zero-field cooling (ZFC) has attracted recent attention due to its interesting physics. In this letter, we report a giant SEB tuned by Sb-doping in Ni_50_Mn_38_Ga_12-x_Sb_x_ Heusler alloys. Such an SEB was switched on below the blocking temperature of approximately 50 K. The maximum exchange bias *H*_*E*_ can arrive at 2930 Oe in a Ni_50_Mn_38_Ga_10_Sb_2_ sample after ZFC to 2 K. Further studies showed that this SEB was attributable to interaction of superspin glass (SSG) and antiferromagnetic matix, which was triggered by the crossover of SSG from canonical spin glass to a cluster spin glass. Our results not only explain the underlying physics of SEB, but also provide a way to tune and control the SEB performance.

Exchange bias (EB) is demonstrated as the magnetic hysteresis loop shifts from the origin, which is usually induced by field cooling (FC) to form a unidirectional magnetic anisotropy at the interface between ferromagnetic (FM) and antiferromagnetic (AFM) phases. The EB effect was discovered in 1956 by Meiklejohn and Bean in Co/CoO nanoparticles^1^, and has been extensively studied and explored in various systems including FM-AFM nanocomposite/bilayers^2^, spin glass (SG)-FM structures^3^, and more^4^,^5^. These systems have significant applications in ultrahigh-density magnetic recording, giant magnetoresistance, and spin valve devices^6^,^7^,^8^. Recently, spontaneous EB (SEB) has attracted much more interest because the exchange bias is able to occur under zero-field cooling (ZFC) conditions. Conventional exchange bias(CEB) is usually observed in systems with interface between different magnetic phases after field cooling, as long as there is net remnant magnetization of the ferromagnetic layer present on cooling to below the blocking temperature. In contrast to CEB, the spontaneous exchange bias(SEB) could be observed without the assistance of external magnetic field. Thus the SEB would be of easy to electric field control of EB devices as it eliminates the requirement of external magnetic field to create the unidirectional anisotropy^9^,^10^. SEB was first reported in NiMnIn alloys by Wang *et al*.^11^ in 2011 and was attributed to a superferromagnetic (SFM) unidirectional anisotropy formed at the interface between superparamagnetic (SPM) domains and the AFM matrix. This SEB was subsequently explored in other magnetic systems and several different mechanisms were proposed. For example, Maity *et al*.^12^ showed SEB in a nanocomposite of BiFeO_3_-Bi_2_Fe_4_O_9_ driven by the superinteraction bias coupling via superspin glass moments at the FM/AFM interface. Han *et al*. observed SEB in a NiMnGa alloy caused by irreversible growth of FM domains changing from a non-percolating to a percolating state^13^. Obviously, a comprehensive understanding of the SEB effect is still disputed.

Of all the materials associated with SEB, off-stoichiometric Mn-rich Ni–Mn–Z (Z = Ga, Sn, In, Sb) Heusler alloys demonstrate remarkable EB owing to their underlying physics^11^,^13^. The Mn-Mn exchange interaction within the regular Mn sublattices and the Mn-Mn exchange interaction between the regular Mn sublattice and the Z sublattice correspond to the FM and the AFM phases, respectively^9^,^14^,^15^. The competition between the FM and the AFM phases can generate frustrated magnetic behavior and thereby lead to the SEB effect. In the other words, these magnetic Heusler alloys firstly undergo a ferromagnetic transition in austenitic state and then a first-order magnetically martensitic transition from an austenitic state with cubic structure to a martensitic state, another magnetization to the spin glass (canonical SG/cluster SG) or the super-paramagnet may occurred when continue to decrease temperature^16^,^17^,^18^. Abundant physical properties could be caused by the SPM or the SG^11^,^13^.

Generally, the research would be more comprehensive through studying of composition-dependent properties. Considering the potential applications, there is an urgent demand for tunable SEB, especially in the read head of ultrahigh sensitivity. In this work, we report a giant SEB effect in Ni_50_Mn_38_Ga_12-x_Sb_x_ Heusler alloys. The maximum exchange bias *H*_*E*_ is 2930 Oe for Ni_50_Mn_38_Ga_10_Sb_2_ at 2 K, which is much higher than most other SEB materials. Most importantly, the *H*_*E*_ can be tuned from 230 Oe to 2930 Oe by adjusting the Sb alloy composition. Based on further analysis and the composition-temperature phase diagram of Ni_50_Mn_38_Ga_12-x_Sb_x_ (where x = 0–6), we demonstrate that this giant SEB is triggered by the crossover of superspin glass from canonical SG to cluster SG.

## Results and Discussions

**Figure 1:**
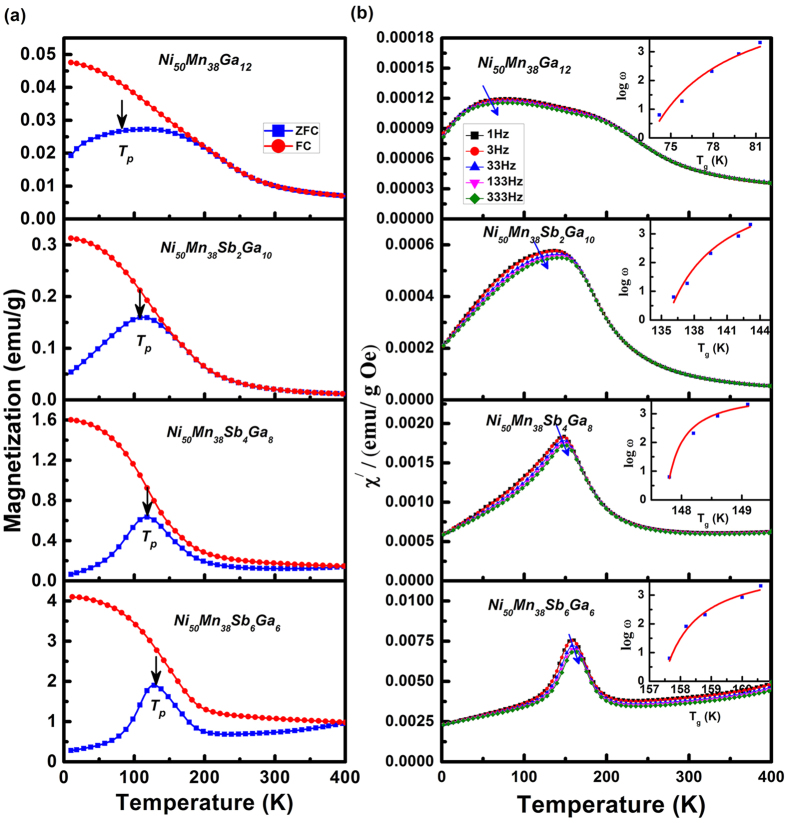
(**a**) The magnetization temperature dependence [M(T)] curves measured at 200 Oe with sequences of ZFC and FC, respectively, (**b**) Temperature dependence of the real part of the ac susceptibility measured at different frequencies with an AC magnetic field of 2 Oe, the inset (**b**) shows the correlation between the angular frequency and the blocking temperature (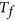). The frequency (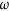) dispersion behavior of temperature (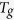) conforms to the Vogel-Fulcher relationship. The arrows indicate the direction of increasing frequencies.

Figure 1(a) shows the magnetization temperature dependence for zero field-cooled (ZFC) and field-cooled` (FC) of Ni_50_Mn_38_Ga_12-x_Sb_x_ (0 ≤ × ≤ 6) alloys under 200 Oe applied magnetic field. Sb0, Sb2, Sb4, and Sb6 correspond to samples Ni_50_Mn_38_Ga_12_Sb_0_, Ni_50_Mn_38_Ga_10_Sb_2_, Ni_50_Mn_38_Ga_8_Sb_4_, and Ni_50_Mn_38_Ga_6_Sb_6_, respectively. It is observed that the magnetization increases as the Sb concentration increases. The ZFC curve exhibits a peak *T*_*p*_ around 100 K and an irreversibility between ZFC and FC curves occurring at *T*_*p*_, which is similar to that of of NiMnIn and NiMnGa^11^,^13^. This peak shifts towards higher temperatures as the Sb concentration increases, as seen at *T*_*p*_ = 75 K, 115 K, 122 K, and 128 K for Sb0, Sb2, Sb4, and Sb6, respectively. Another interesting feature is the systematic difference between the ZFC and the FC curves, with an appearance of an irreversible behavior below the *T*_*p*_. In general, the M-T behaviors of the SG and the SPM systems at lower temperature are similar under zero field cooling. However, when cooled in the presence of a magnetic field, the magnetization increases monotonically in SPM systems as the temperature decreases. Rather, the magnetization of SG systems tends to saturate or decrease with decreasing temperature. As shown in Fig. 1(a), the FC curve behavior of all samples indicates the SG feature in the alloys.

To further clarify the origin of the complicated magnetic states below *T*_*P*_, the temperature dependence of the real portion (χ^/^) of the AC susceptibility was measured at a low magnetic field strength of 2 Oe (Fig. 1b). The peak shifted toward higher temperatures and its value decreased as frequency increased. The shape of the peak at *T*_*P*_ became distinct for increased Sb concentration. This may be attributed to the atomic radius of Sb, which is larger than that of Ga; a larger atomic radius weakens the AFM by increasing the Mn-Mn distance.

To extract additional parameters, we use the Vogel-Fulcher(V-F) relationship to analyze the SG-like behavior^19^.


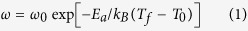


where *ω* is the measurement angular frequency, 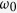 is the characteristic frequency of the SG, *E*_*a*_ is the activation energy of the SG, *k*_*B*_ is the Boltzmann constant, *T*_*f*_ is the frequency temperature defined above, and *T*_*0*_ is the V-F temperature that describes the interaction among SG clusters. The *T*_*0*_ values are very close to the *T*_*P*_ values obtained from the MT curves at 200 Oe in Fig. 1(a). *τ*_0_ (*τ*_0_ = 2*π*/*ω*_0_) is the microscopic relaxation time related to spin glass or cluster spin glass. All of the parameters were obtained by best fit and are shown in Table 1. These SG observations are in good agreement with results from the literature. For example, *τ*_0_ varies from 10^−11^~10^−15^ for canonical SG, but the typical value for cluster SG compounds is around *τ*_0_ = 10^−919,^^20^. It is obvious from Table 1 that all the characteristic parameters fall in the typical value ranges.

**Figure 2:**
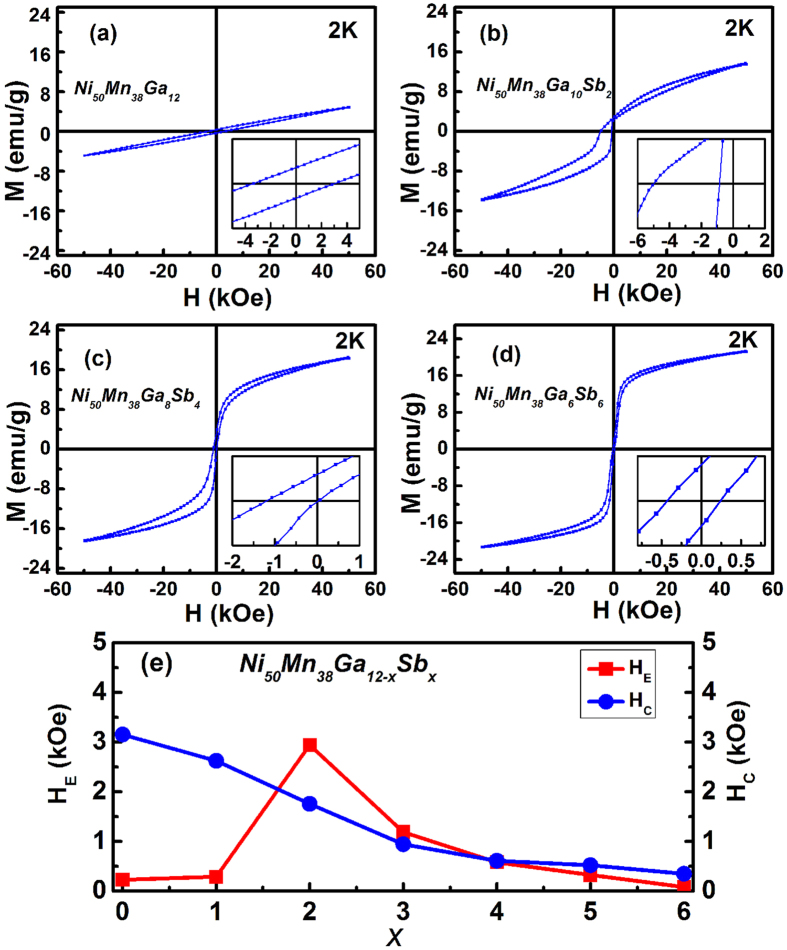
(**a**–**d**) Magnetic hysteresis loops for Ni_50_Mn_38_Ga_12-x_Sb_x_ (x = 0, 2, 4, and 6) samples measured at 2 K after zero field cooling. (**e**) *H*_*E*_ and *H*_*C*_ dependent on the Sb content of Ni_50_Mn_38_Ga_12-x_Sb_x_ (x = 0–6) measured at 50 kOe after ZFC.

According to previous reports^11^,^14^, the SEB normally occurs in complex magnetic states of SG, AFM, and SSG. Hence, the magnetization loops M(H) for different Ni_50_Mn_38_Ga_12-x_Sb_x_ alloys have been measured at 2 K, as indicated in Fig. 2(a–d). The inset of each figure shows a magnified view of the loop shift towards the negative field axis; a pronounced shift can be seen. All the hysteresis loops are shifted along the field axis, indicating the presence of the EB effect. Note that the sample was cooled in a zero magnetic field from 400 K.

The FM characteristic is more evident as Sb concertation increases, which indicates that the AFM interaction decreases as well. A clear SEB effect can be found at x = 2 and x = 4 (inset figure), but is absent at x = 0 and x = 6. The largest EB is observed at x = 2. This phenomenon may be attributed to the fact that Ni_50_Mn_38_Ga_10_Sb_2_ is a boundary of canonical spin and cluster spin. At the phase boundary, the ferroic system thermodynamically achieves a state with a flattened free energy profile, in which the polarization or magnetization can easily rotate almost without energy barrier. *H*_*E*_ and *H*_*C*_ are defined as *H*_*E*_ =  −(*H*_*L*_ + *H*_*R*_)/2 and *H*_*C*_ = −(*H*_*L*_ − *H*_*R*_)/2, respectively, where *H*_*L*_ and *H*_*R*_ are the left and right coercive fields, respectively^6^,^8^. *H*_*E*_ and coercivity *H*_*C*_ as functions of Sb content are shown in Fig. 2(e) for the different Ni_50_Mn_38_Ga_12-x_Sb_x_ alloys. The *H*_*E*_ and *H*_*C*_ change from 230 Oe and 2930 Oe to 610.83 Oe and 1024.16 Oe, respectively, as the Sb concentration increases from 0 to 2. The *H*_*E*_ varies in the 2930 Oe and 80 Oe range upon doping at x = 6, and the coercivity (*H*_*C*_) also decreases from 2070 to 350 Oe.

**Figure 3:**
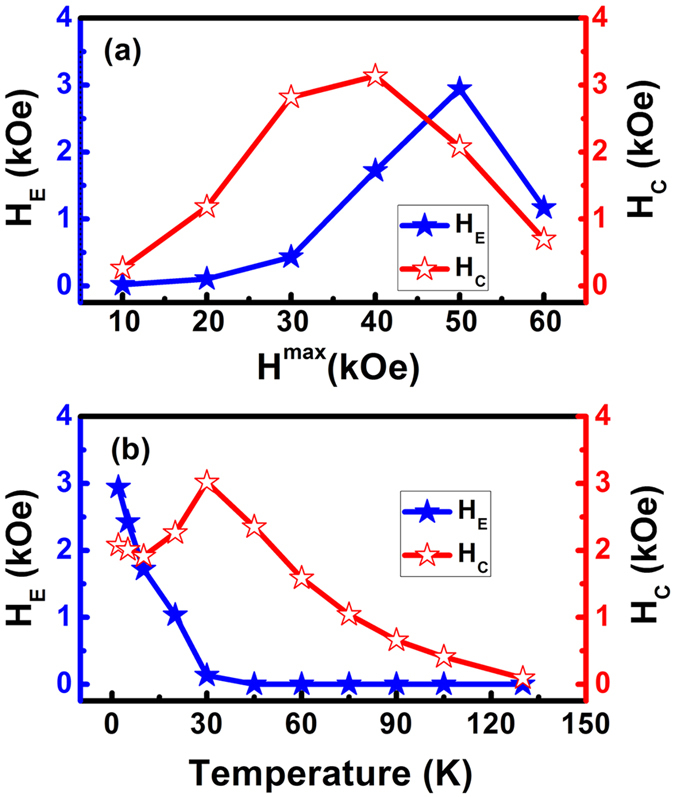
(**a**) *H*_*E*_ (blue) and *H*_*C*_ (red) as a function of *H*_*max*_ in Ni_50_Mn_38_Ga_10_Sb_2_ at 2 K after ZFC. (**b**) *H*_*E*_ (black) and *H*_*C*_ (red) as a function of temperature in Ni_50_Mn_38_Ga_10_Sb_2_ after ZFC.

The EB effect for the Ni_50_Mn_38_Ga_10_Sb_2_ alloy at different magnetic fields (10 kOe to 60 kOe at 2 K) was also investigated. As shown in Fig. 3(a), *H*_*E*_ is 100 Oe when the applied magnetic field is under 20 kOe but continually grows until reaching a maximum value of 2930 Oe at the critical magnetic field of 50 kOe. This maximum occurring at the field of 50 kOe suggests the formation of the maximum SSG unidirectional anisotropy. Beyond an applied magnetic field strength of 50 kOe, the *H*_*E*_ begins to decrease. The *H*_*E*_ decrease may originate from the change of the SSG spin structure under larger applied magnetic fields. Meanwhile, *H*_*C*_ shows a non-monotonic behavior. The maximum of *H*_*C*_ occurs at 40 kOe, which is lower than that of *H*_*E*_. This indicates that interfaces associated with phase separation are not so stable in high magnetic fields because of the propagation of the SSG phase. A high magnetic field can disturb the interfacial exchange coupling and remove the exchange anisotropy, which can lead to the EB disappearing. From these observations, we can conclude that the number of SPM domains in the SSG state will decrease with increasing *H* due to more of the SSG states transforming to AFM states. This generates new interfaces with SSG unidirectional anisotropy^11^. The calculated *H*_*E*_ and *H*_*C*_ as a function of temperature for the Ni_50_Mn_38_Ga_10_Sb_2_ alloy are depicted in Fig. 3(b). *H*_*E*_ decreases as the temperature increases, and becomes almost zero at about 50 K. This temperature can be referred to as the EB blocking temperature (*T*_*B*_), because the EB effect vanishes above this temperature; it can be attributed to the weakening of the SSG-AFM coupling as temperature increases. The disappearance of the *H*_*E*_ value above the blocking temperature (*T*_*B*_ ≈ 50 K) is due to the fact that AFM interactions begin to dominate the SSG interactions. On the other hand, *H*_*C*_ initially increases with temperature and begins to decrease after reaching a maximum value. It is known that anisotropy of SSG decreases with increasing temperature, because the AFM is able to drag more SSG spins resulting in increasing coercivity below *T*_*B*_.

**Figure 4:**
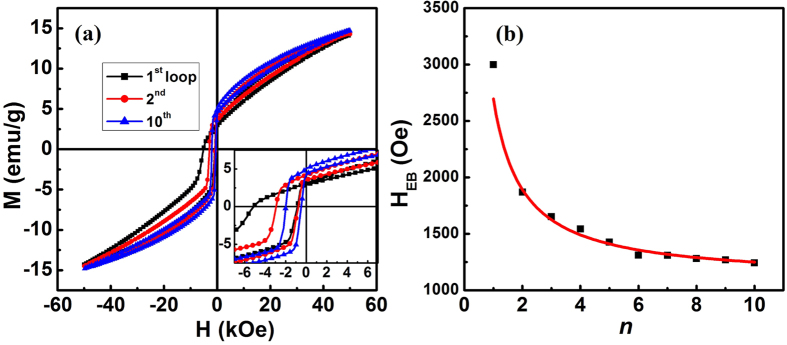
(**a**) Training effect of sample Ni_50_Mn_38_Ga_10_Sb_2_. The inset shows enlarge of hysteresis loop. (**b**) Cycle dependence of *H*_EB_ of the Ni_50_Mn_38_Ga_10_Sb_2_. The solid line shows the best fit of Equation (2) to the data for *n* *>* 1.

To investigate the SEB training effect, the sample was cooled without field from 400 K to 2 K and ten hysteresis loops were measured, consecutively. As plotted in Fig. 4, training effect is obviously observed in Ni_50_Mn_38_Ga_10_Sb_2_ alloy. The first cycle loop shows a prominent SEB effect with an asymmetric magnetization reversal characteristic. After that, the subsequent magnetization cycles tend to symmetric gradually. The central part of first, second, and tenth loops are shown in inset Fig. 4(a). As seen, the left coercive field *H*_*L*_ decreases dramatically with increased cycle of n, while *H*_*R*_ shows a qualitatively similar but by far less pronounced dependence on *n*. The different strengths of *n* dependences of *H*_*L*_ and *H*_*R*_ indicate that the left and right branches of the hysteresis follow different mechanisms of magnetization reversal. The usual experimentally observed relationship between *H*_*EB*_ and *n* can be expressed by equation (2).


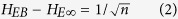


where *H*_*E*∞_ is the exchange-bias field at the *n*th cycle (in the limit of an infinite number of cycles). The results presented in Fig. 4(b) were fitted using the Equation (2), giving *H*_*E*∞_ = 1210 Oe. This training effect was interpreted in terms of metastable magnetic disorder at the magnetically frustrated interface during the magnetization reversal process for SEB^21^,^22^.

**Figure 5:**
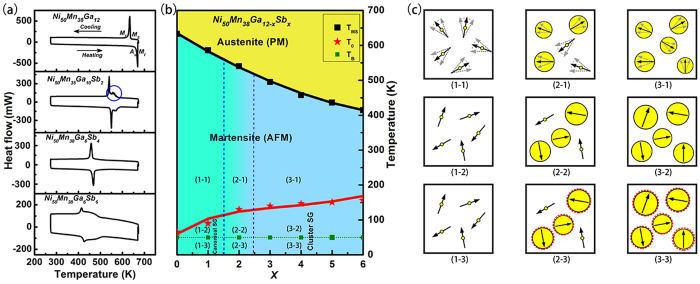
(**a**) The martensitic transformation behavior of Ni_50_Mn_38_Ga_10_Sb_2_ measured with DSC at a cooling/heating rate 20 K/min. (**b**) Composition-temperature phase diagram of Ni_50_Mn_38_Ga_12-x_ Sb_x_ (x = 0–6) alloys. (**c**) Simplified schematic diagrams of the domain evolution embedded in an AFM single domain and hysteresis loops at different temperature.

To further detail composition-dependent SEB evolution, we constructed the composition-temperature phase diagram of Ni_50_Mn_38_Ga_12-x_Sb_x_ (where x = 0–6). The composition-temperature phase diagram was determined by the martensite start temperature, *T*_*MS*_, detected using DSC [Fig. 5(a)], the spin glass temperature, *T*_*0*_, determined by fitting AC lines [Fig. 1(b)], and the block temperature, *T*_*B*_, collected by measuring the exchange bias (*H*_*E*_) versus temperature (*T*). Figure 5(a) shows the DSC of the alloys, measured at 10 K/min heating and cooling rates. Large exothermic and endothermic peaks during cooling and heating were observed corresponding to direct and reverse martensitic transformations, respectively. Take Ni_50_Mn_38_Ga_12_ as an example, its characteristic martensitic transition temperatures including the austenite start and finish (*A*_*s*_ and *A*_*f*_) and martensite start and finish (*M*_*s*_ and *M*_*f*_) temperatures are 660 K, 674 K, 636 K, and 620 K, respectively. Its corresponding thermal hysteresis can become as large as 20 K, indicating that the martensitic phase transition is a first order phase transition. It also can be seen that the increase of Sb content may shift the transformation peaks to a lower temperature regime. Therefore, a partial substitution of Ga by Sb would suppress the martensitic transformation occurrence. In particular, we observed a wild martensitic transformation behavior in the DSC curve of the Ni_50_Mn_38_Ga_10_Sb_2_ alloy [see Fig. 5(a) blue circle]. This DSC peak characteristic probably account for the presence of jerky characteristic in martensitic phase of the Ni_50_Mn_38_Ga_10_Sb_2_ alloy. It would be shown some special performance in martensitic phase^23^,^24^,^25^. This is in good agreement with previous AC curves (Fig. 1). In addition, the composition Ni_50_Mn_38_Ga_10_Sb_2_ might be considered as co-existence of canonical spin and cluster spin, with the cluster spin being dominant.

From the phase diagram shown in Fig. 5(b), the martensite transformation temperature of the Ni_50_Mn_38_Sb_x_Ga_12-x_ (x = 0–6) alloys decreases slightly as the Sb content increases. At the same time, the AFM phase remains almost unchanged while the SG phase varies with the Sb content. In addition, the pinning phase changes from canonical SG to cluster SG when *x* is larger than 3. The *T*_*B*_ holds at 50 K, because the anisotropy of SG is more related to temperature. We also considered the evolution of the magnetic state of Ni_50_Mn_38_Ga_12-x_Sb_x_ (x = 0–6) after ZFC under different temperatures, as shown in the inset of Fig. 5(b). Regions (1), (2), and (3) corresponds to (x ≤ 1), (1 <× < 3), and (3 ≤ × ≤ 6), respectively. The physical schematic diagram for the martensitic phase is shown in Fig. 5(1–1),(2–1) and (3–1); it is a simplified schematic diagram with unfrozen nano SPM domains embedded in an AFM single domain under martensitic phase. The domain sizes increase with the increase of Sb constant. Moreover, all the domains are random. Upon cooling to *T* < *T*_*0*_, the thermal energy becomes lower than the average energy barrier for flipping local domains. As a result, the domain flipping essentially stopped and the system is frozen. This process represents a glass transition from the martenstic state to a frozen spin glass state. The physical schematic diagram for the SG system is drawn in Fig. 5(1–2),(2–2) and (3–2). The domain size is larger than that in martensitic phase, and the domain is frozen. The part of (1–2) shows canonical SG and the part (3–2) shows the cluster SG. Therefore, the Ni_50_Mn_38_Ga_10_Sb_2_ alloy can be regarded as a boundary between the canonical SG and the cluster SG. It shows the cluster SG (calculate by fitting AC lines), but it also keeps some canonical SG domain. As the temperature further decreases to *T* < *T*_*B*,_ the anisotropy in the SG phase increases [see dashed red circles in (2–3), (3-3)]. More important, at the phase boundary, the system achieves a thermodynamic state with a flattened free energy profile. In this state, the polarization or magnetization is easily rotated almost without energy barrier^26^,^27^,^28^. Therefore, the largest *H*_*E*_ (~2930 Oe) was obtained in the Ni_50_Mn_38_Ga_10_Sb_2_ alloy sample.

## Conclusion

In summary, SEB continuous tuning was demonstrated in Ni_50_Mn_38_Ga_12-x_Sb_x_ (x = 0–6) alloys, and the largest *H*_*E*_ (~2930 Oe) was obtained in the Ni_50_Mn_38_Ga_10_Sb_2_ sample. Moreover, the tunable *H*_*E*_ physical mechanism was investigated. This behavior can be explained by the pinning phase from the canonical SG to the cluster SG through the change of the atomic radius between Mn-Mn by substituting Z under the off-stoichiometric Mn rich Ni-Mn-Z (Z = Sn, Ga, and Sb) alloys. The Ni_50_Mn_38_Ga_10_Sb_2_ alloy is like a phase boundary between the canonical SG and the cluster SG at lower temperatures. Thus, the largest SEB is observed in the Ni_50_Mn_38_Ga_10_Sb_2_ sample. More remarkable, the SEB decreased with increasing temperature, approaching zero around *T *= 50 K. The SEB change is due to decreasing SG phase anisotropy [see the dashed red circles in Fig. 5(b)] as temperature increases. Our results not only open a new direction to realize the EB effect, but also indicate a way to fabricate SEB with different values and promote the application of magnetic Heusler alloys in the ultrahigh-density magnetic recording, giant magnetoresistance, and spin-valve devices.

## Methods

The polycrystalline Ni_50_Mn_38_Ga_12-x_Sb_x_ (x = 0~6) alloys with are prepared by arc melting high-purity (99.99%) Ni, Mn, Ga, and Sb in an argon atmosphere. To achieve high composition homogeneity, the samples are wrapped in silica tubes and are vacuum annealed at 1173 K for 24 hours. We used differential scanning calorimetry (DSC) to determine the martensitic transition temperature. The magnetic properties, including magnetic hysteresis (MH) loops, magnetization-temperature (MT) curves, and AC susceptibility measurements are measured using a superconducting quantum interference device (SQUID) magnetometer (Quantum Design, MPMS-XL-5). We used the function of magnet reset in SQUID (A heater integrated into the magnet solenoid may be used to drive the wire above its critical temperature and eliminate most trapped magnetic flux.) to demagnetize the superconducting magnet before testing ZEB effect, and all the samples were first warm to 400 K and then cooled to 2 K before test. The composition-temperature phase diagram of Ni_50_Mn_38_Ga_12-x_Sb_x_ (x = 0–6) is plotted according to the magnetic properties and the EB measurements.

## Additional Information

**How to cite this article**: Tian, F. *et al*. Giant spontaneous exchange bias triggered by crossover of superspin glass in Sb-doped Ni_50_Mn_38_Ga_12_ Heusler alloys. *Sci. Rep.* **6**, 30801; doi: 10.1038/srep30801 (2016).

## Figures and Tables

**Table 1:** Parameter values obtained by fitting the experimental data to Equation (1).

Samples	T_0_(K)	τ_0_(s)	E_a_/K_B_(K)	
**Ni**_**50**_**Mn**_**38**_**Ga**_**12**_	65.1	9.3 × 10^−14^	43.8	canonical spin glass
**Ni**_**50**_**Mn**_**38**_**Ga**_**11**_**Sb**_**1**_	90.3	8.7 × 10^−12^	72.5	canonical spin glass
**Ni**_**50**_**Mn**_**38**_**Ga**_**10**_**Sb**_**2**_	130.7	2.4 × 10^−10^	104.1	cluster spin glass
**Ni**_**50**_**Mn**_**38**_**Ga**_**9**_**Sb**_**3**_	140.4	5.3 × 10^−9^	158.4	cluster spin glass
**Ni**_**50**_**Mn**_**38**_**Ga**_**8**_**Sb**_**4**_	147.5	7.2 × 10^−9^	173.3	cluster spin glass
**Ni**_**50**_**Mn**_**38**_**Ga**_**7**_**Sb**_**5**_	151.9	2.1 × 10^−8^	191.7	cluster spin glass
**Ni**_**50**_**Mn**_**38**_**Ga**_**6**_**Sb**_**6**_	156.4	1.3 × 10^−7^	213.6	cluster spin glass
